# Crystal structure of ChbG from *Klebsiella pneumoniae* reveals the molecular basis of diacetylchitobiose deacetylation

**DOI:** 10.1038/s42003-022-03824-9

**Published:** 2022-08-24

**Authors:** So Yeon Lee, Bashu Dev Pardhe, Tae-Jin Oh, Hyun Ho Park

**Affiliations:** 1 College of Pharmacy, Chung-Ang University, Seoul, 06974 Republic of Korea; 2 Department of Global Innovative Drugs, Graduate School of Chung-Ang University, Seoul, 06974 Republic of Korea; 3 Department of Life Science and Biochemical Engineering, Sunmoon University, Chungnam, 31460 Republic of Korea; 4 Department of Pharmaceutical Engineering and Biotechnology, Sunmoon University, Chungnam, 31460 Republic of Korea; 5 Genome-based BioIT Convergence Institute, Chungnam, 31460 Republic of Korea

**Keywords:** X-ray crystallography, Enzymes

## Abstract

The chitobiose (*chb*) operon is involved in the synthesis of chitooligosaccharide and is comprised of a *BCARFG* gene cluster. ChbG encodes a chitooligosaccharide deacetylase (CDA) which catalyzes the removal of one acetyl group from N,N’-diacetylchitobiose. It is considered a novel type of CDA due to its lack of sequence homology. Although there are various structural studies of CDAs linked to the kinetic properties of the enzyme, the structural information of ChbG is unavailable. In this study, the crystal structure of ChbG from *Klebsiella pneumoniae* is provided. The molecular basis of deacetylation of diacetylchitobiose by ChbG is determined based on structural analysis, mutagenesis, biophysical analysis, and in silico docking of the substrate, diacetylchitobiose. This study contributes towards a deeper understanding of chitin and chitosan biology, as well as provides a platform to engineer CDA biocatalysts.

## Abstract (web summary)

Structural and functional characterization of *Klebsiella pneumonia* ChbG (which lacks sequence homology) reveals the mechanism of chitooligosaccharide processing by ChbG.

## Introduction

Chitin is a linear polysaccharide polymer of β-(1–4)-linked *N*-acetyl-glucosamine (GlcNAc) units found in mollusk shells, insect exoskeletons, and fungal cell walls^1,2^. Its depolymerization and deacetylation produces various polysaccharide derivatives such as chitosan and chitooligosaccharide which are critical elements for cellular functions including cell-recognition, immune response, and morphogenesis^3–5^. Many species including bacteria use chitin-derived chitooligosaccharide as an energy source for survival^6^.

Chitin deacetylation is catalyzed by different classes of chitin deacetylases (CDAs) with variable substrate specificity^7^. CDAs and chitooligosaccharide deacetylases (CODs) belong to the carbohydrate esterase family 4 (CE4) that contains a conserved NodB domain^8,9^. More recently, CE14 family CODs were characterized from archaea and *Bacillus* species based on sequence similarity^10,11^. The industrial application of CDAs includes the production of various chitin-derived bioactive molecules and potential drug targets against pathogenic microorganisms^12,13^.

The chitobiose operon (*chb*-*BCARFG*) from *Escherichia coli* is activated to utilize chitooligosaccharide when it is the sole carbon source^14^. ChbG is a COD that catalyzes the removal of an acetyl group from the chitooligosaccharide, N,N’-diacetylchitobiose to yield N-acetyl-β-glucosaminyl-glucosamine^14,15^. This deacetylase can also use diacetylchitobiose-6-phosphate as a substrate to produce monoacetylchitobiose-6-phosphate, which is the inducer and substrate of ChbR and ChbF, respectively^14^. ChbG is a “non-classified” carbohydrate esterase in the Carbohydrate Active Enzymes (CAZY) database due to the lack of sequence similarity with other CDAs and CODs^9^.

In this study, a model of the molecular basis of diacetylchitobiose deacetylation is presented by determining the high-resolution crystal structure of ChbG from *Klebsiella pneumoniae* (hereafter called kpChbG). We initially sought to use ChbG from E.coli (ecChbG) for structural and enzymology studies because it is the most studied ChbG in the chitobiose operon; however, we could not properly produce ecChbG in our bacterial expression system. Thus, we examined ChbG in other bacteria and found that kpChbG was the most soluble among seven tested ChbG homologs and it purified sufficiently well for structural and biochemical studies. Our study will deepen the understanding of chitin and chitosan biology, as well as help to engineer CDAs and CODs as biocatalysts to produce various chitosan products for industrial and medicinal applications.

## Results and discussion

### Overall kpChbG structure

**Fig. 1:**
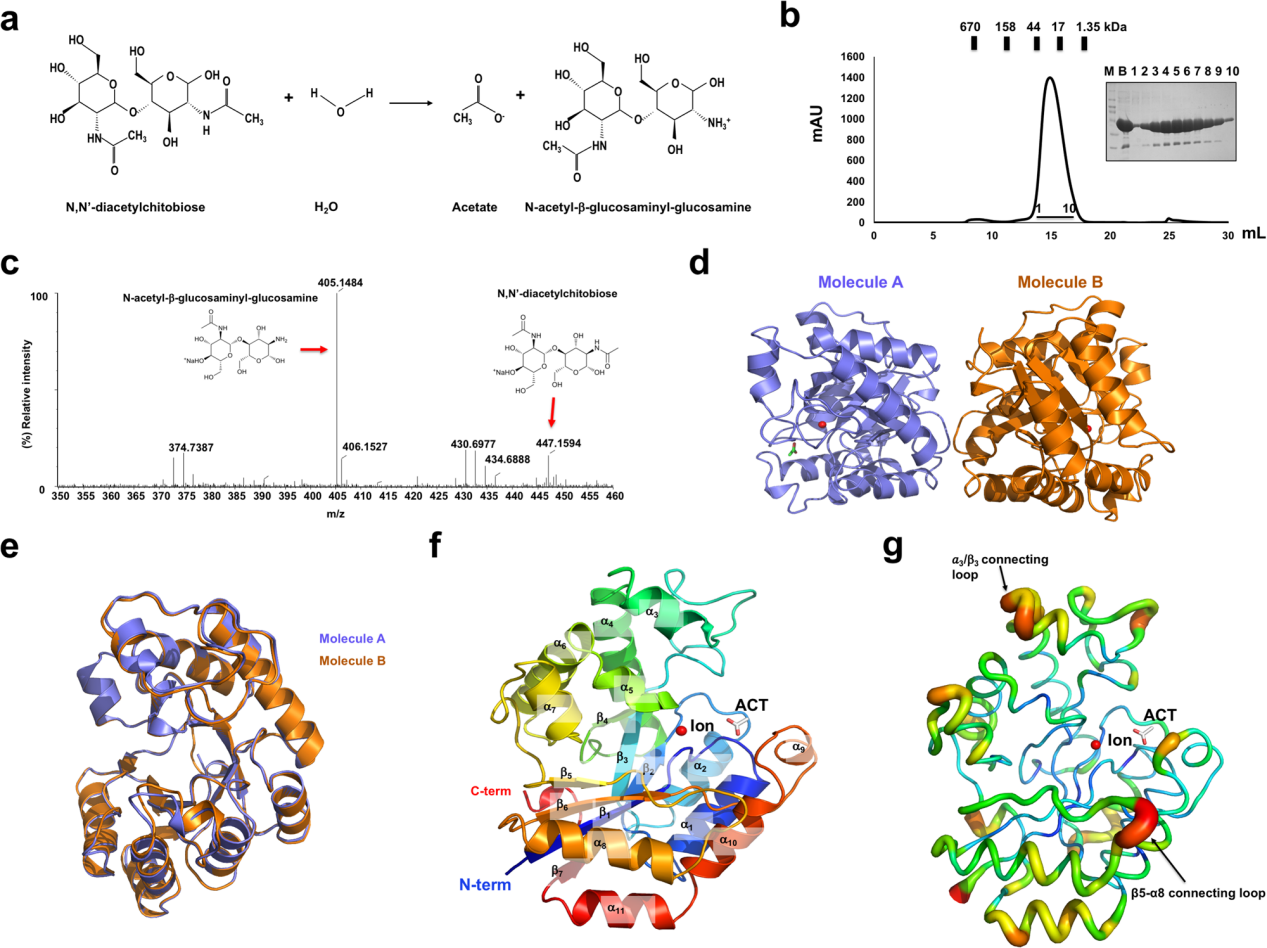
Crystal structure of ChbG from Klebsiella pneumoniae (kpChbG). **a** Catalytic reaction of ChbG. **b** Size-exclusion chromatography (SEC) of Ni^2+^-affinity purified ChbG. Loaded fractions are indicated by the horizontal black bar. Inset: SDS-PAGE assessment of purity. M: size marker and B: before SEC loading. **c** ESI-MS analysis of deacetylation of N,N’-diacetylchitobiose ((GlcNAc)_2_) to N-acetyl-β-glucosaminyl-glucosamine (GlcN-GlcNAc) by kpChbG. **d** Cartoon representation of two kpChbG molecules (molecule A and B) presented in an asymmetric unit. **e** Superposition of the structures in one asymmetric unit. **f** Rainbow-colored cartoon representation of monomeric kpChbG. The polypeptide chain from the N-terminus to the C-terminus is colored blue to red. Helices and sheets are labeled α and β, respectively. ACT represents acetate and Ion is the metal ion. **g** Putty representation showing *B*-factor distribution in the order of *B*-factor values using rainbow colors (red to violet).

Full-length kpChbG protein was purified using a quick two-step chromatography procedure involving Ni^2+^-affinity followed by size exclusion chromatography (SEC). Elution at approximately 15 mL on the SEC column indicated that kpChbG formed a dimer in solution (Fig. 1b and Supplementary Fig. 1). Before structural analysis, we measured the deacetylation activity of kpChbG using electrospray ionization mass spectrometry (ESI-MS) to ensure that the purified enzyme was active and ChbG from different species is real ChbG enzyme. These measurements showed that purified kpChbG exhibited significant deacetylation activity using N,N’-diacetylchitobiose as substrate, producing N-acetyl-glucosaminyl-glucosamine (Fig. 1c and Supplementary Fig. 2). Thereafter, the purified protein was crystallized, and the 1.83 Å crystal structure was solved using the molecular replacement phasing method and refined to R_work_ = 20.24 % and R_free_ = 25.02 %. The previously deposited but unpublished structure of the YdjC family (PDB ID: 2I5I) has 35 % amino acid sequence homology with kpChbG and was used as the search model. The crystallographic and refinement statistics are summarized in Table 1.

**Table 1:** Data collection and refinement statistics.

**Data collection**	
Space group	*P2*_*1*_*2*_*1*_*2*_*1*_
Unit cell parameter *a*, *b*, *c* (Å)	*a* = 54.23, *b* = 67.03, *c* = 146.40
*α, β, γ* (°)	*α* = 90, *β* = 90, *γ* = 90
Resolution range (Å)^a^	28.51–1.83
Total reflections	574,384
Unique reflections	47,466
Multiplicity	12.10 (10.78)
Completeness (%)^a^	99.05 (99.59)
Mean *I*/σ(*I*)^1^	15.00 (1.50)
*R*_merge_^a,b^	0.10 (1.96)
Wilson *B*-factor (Å^b^)	24.11
**Refinement**	
Resolution range (Å)	28.51–1.83
Reflections	47,456
*R*_work_ (%)	20.24 (29.73)
*R*_free_ (%)	25.02 (34.41)
No. of molecules in the asymmetric unit	2
No. of non-hydrogen atoms	4285
Non-solvent	3897
Solvent	388
Average *B*-factor values (Å^2^)	27.77
Non-solvent	26.79
Solvent	34.27
Ramachandran plot:	
Favored / allowed / outliers (%)	97.40 / 2.60 / 0.00
Rotamer outliers (%)	0.25
Clashscore	2.87
RMSD bonds (Å) / angles (°)	0.006 / 0.822

^a^Values for the outermost resolution shell in parentheses.

^b^R_merge_ = Σ_h_ Σ_i_ | *I*(*h*)_i_ − <*I*(*h*)>|/ Σ_*h*_ Σ_*i*_ I(*h*)_*i*_, where *I*(*h*) is the observed intensity of reflection h, and <*I*(*h*)> is the average intensity obtained from multiple measurements.

Two molecules (A and B) were found in the crystallographic asymmetric unit (Fig. 1d). The models of both molecules constructed from residue 2 to residue 252 were nearly identical with a root-mean-square deviation (RMSD) of 0.3 Å (Fig. 1e). The kpChbG crystal structure showed the typical fold of the YdjC family and contained an unbalanced α/β barrel fold consisting of eleven α-helices (α1–α11) and seven β-sheets (β1–β7) (Fig. 1f). An acetate molecule and metal ion were found in the putative active site of kpChbG (Fig. 1f). *B*-factor analysis showed that the kpChbG structure contained two high *B*-factor regions: the α3-α4 connecting loop and the β5-α8 connecting loop (Fig. 1g). However, the *B*-factor of the tentative active site with the acetate ion and metal ion was low, indicating that this region was rigid in solution (Fig. 1g).

### kpChbG forms a dimer in solution

**Fig. 2:**
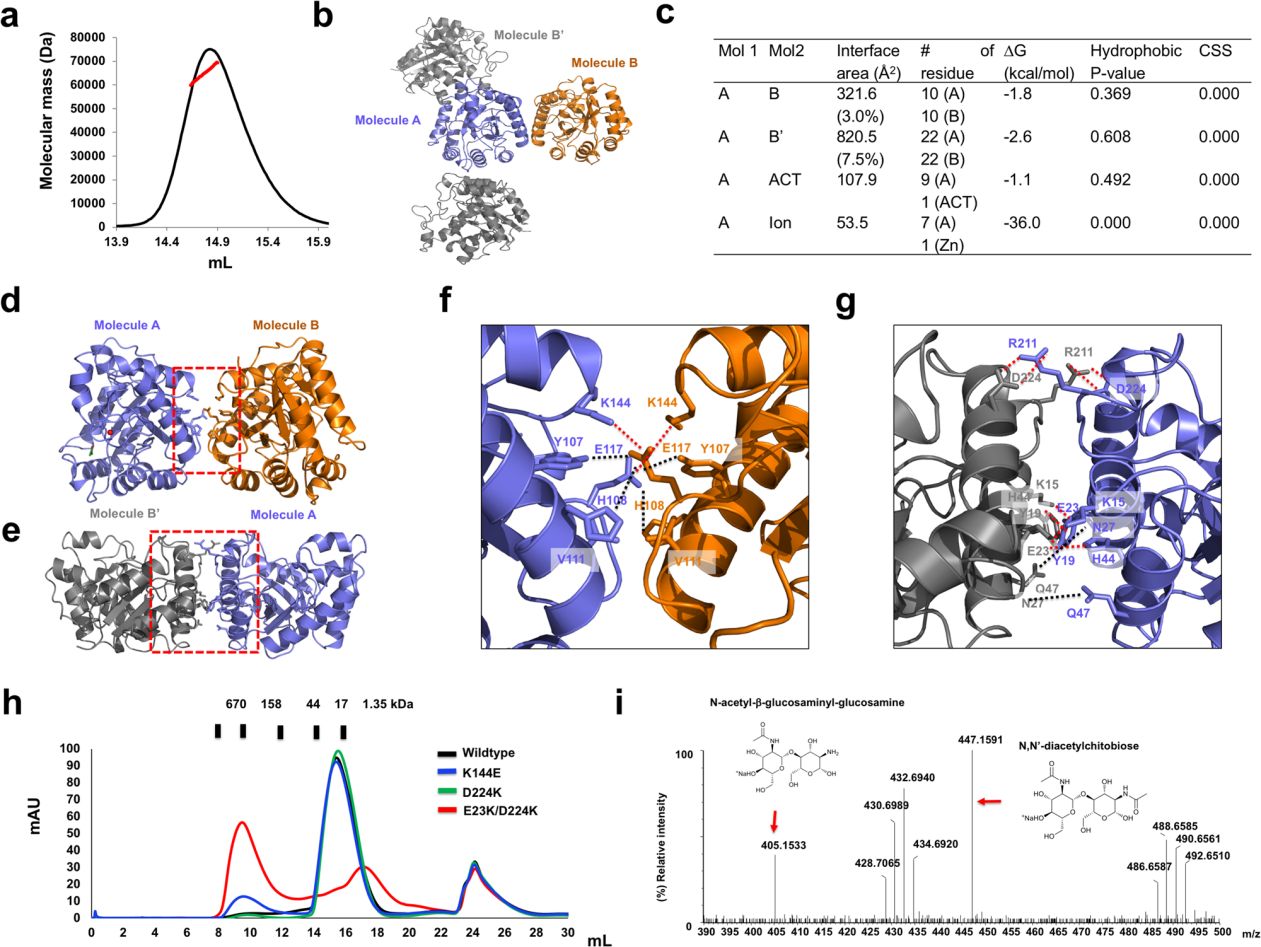
Dimeric structure of kpChbG. **a** Multi-angle light scattering (MALS) profile of the SEC eluted kpChbG peak. The red line indicates the experimental molecular mass analyzed by MALS. **b** Crystallographic packing symmetry analyzed by PyMOL. Two kpChbG molecules (A and B) found in the asymmetric unit are colored in blue and orange ribbons, while the other symmetric molecules are colored gray. **c** Table summarizing the interaction details of each type of interface analyzed by the PISA server. ACT and CSS indicate acetate and the complex formation significance score, respectively. Cartoon representation of the putative dimeric structure of the A/B complex (**d**) and the A/B’ complex (**e**). The red-dashed lines of the A/B complex and the A/B’ complex represent the magnified region of the interfaces shown in **f** and **g**, respectively. Red-dashed lines and black-dashed lines indicate salt bridges and hydrogen bonds, respectively. Residues that are involved in dimer formation are labeled. **h** SEC profiles of wild type kpChbG and mutant kpChbG at putative interface binding sites. **i** ESI-MS analysis of deacetylation of N,N’-diacetylchitobiose to N-acetyl-β-glucosaminyl-glucosamine by the dimer disruption mutant, E23K/D224K.

A dimeric YdjC-family protein was previously suggested, although there is no direct evidence^16^. The theoretical molecular weight of monomeric kpChbG, including the C-terminal his-tag is 30.2 kDa. Multi-angle light scattering (MALS) showed that the absolute molecular mass of kpChbG in solution was 64.9 kDa (2.1 % fitting error) (Fig. 2a), which confirmed that kpChbG is a dimer in solution.

The dimeric interface analyzed by crystallographic packing found a putative alternative dimeric structure formed between molecule A and symmetry molecule B’ (Fig. 2b). Two types of dimers (A/B dimer or A/B’dimer) might be the meaningful real dimer, so the protein-protein interactions (PPI) in both the A/B dimer and the A/B’ dimer were further analyzed using the PDBePISA PPI-calculating server (Fig. 2c)^17^. The complex formation significance score (CSS) of both dimers was zero, indicating that they may not form in solution, which contradicted the SEC-MALS result. A total of 20 residues (10 from each molecule) were involved in PPI of the A/B dimer, whose total surface buried an area of 321.6 Å^2^, representing 3.0% of the total surface area (Fig. 2c, d). Meanwhile, 44 residues (22 from each molecule) were involved in the PPI of the A/B’ dimer (Fig. 2c, e), whose total surface buried an area of 820.5 Å^2^, representing 7.5% of the total surface. The main forces maintaining the A/B dimer were massive salt bridges and hydrogen-bonds formed by Y107, H108, E117, and K114 from both molecules (Fig. 2f). The A/B’ dimer was maintained by forces from two distinct interface regions: salt bridges generated by R211 and D224 from each molecule (Fig. 2g), and massive salt bridges and hydrogen-bonds generated by K15, Y19, E23, N27, H44, and G47 from each molecule (Fig. 2g). Therefore, we suggested that this dimer was the more likely to be formed in solution. Mutagenesis of the critical residues for the formation of the putative A/B dimer (K114) and A/B’ dimer (D224K) failed to disrupt dimer formation, since the SEC elution times were the same as the wild type (Fig. 2h). Mutation of the amino acid (E23K) critical for the second interface of the putative A/B’ dimer to produce the E23K/D224K double-mutant resulted in its elution at approximately 17 mL on the SEC column. This indicated that the double mutant disrupted both PPI regions of the A/B’ dimer to produce a monomer and supported the initial suggestion that the A/B’ dimer of kpChbG is formed in solution. The E23K/D224K double-mutant also generated a detectable peak at approximately 8 mL on the SEC column (Fig. 2h). Sodium dodecyl sulfate polyacrylamide gel electrophoresis (SDS-PAGE) analysis of the eluted peak revealed that it was the E23K/D224K double-mutant ChbG protein (Supplementary Fig 3). The earlier elution of the double-mutant around the void volume might be due to the loss of solubility of ChbG as a result of mutagenesis. Although the solubility of kpChbG decreased after introducing double-mutations, a detectable amount of soluble kpChbG protein was detected at the size factions corresponding with the monomer.

To understand the functional implication of kpChbG dimerization, we measured the deacetylation activity of the dimerization disrupting mutant E23K/D224K. As shown in Fig. 2i, the activity of the E23K/D224K mutant was dramatically reduced more than 50% compared with wildtype activity, indicating that dimerization of kpChbG affects the full deacetylation activity of ChbG.

### Metal ion and product coordination in the kpChbG structure

**Fig. 3:**
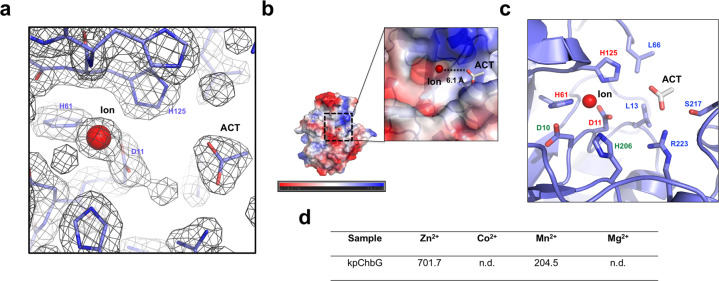
Identification of the Zn2+ (Ion) and acetate (ACT) ion in the putative active site of kpChbG. **a** The electron density map around the metal ion binding site of kpChbG. The 2Fo-Fc map contoured at the 1σ level is shown. Residues coordinated with the metal ion are labeled. **b** Electrostatic surface representation of kpChbG. The scale bar ranges from −7.3 kT/e (red) to 7.3 kT/e (blue). The black dashed box shows the magnified region of the putative active site. **c** Cartoon representation of the putative active site of kpChbG. Residues involved in coordination of the metal ion and acetate ion are colored red and blue, respectively. Critical residues involved in kpChbG catalysis (D10 and H206) are colored green. **d** Table showing the metal ion concentration in kpChbG (µg/kg) analyzed by ICP-MS. n.d.: not determined.

Various CDAs and CODs require metal ions such as Zn^2+^, Mn^2+^, Co^2+^, and Mg^2+^ for deacetylation activity^16,18,19^. Clear metal ion density coordinated by D11, H61, and H125 was detected at the putative active site of kpChbG (Fig. 3a). A bulb-like density was also detected at the putative active site near the metal ion density (Fig. 3a). We believe that this bulb-like density was produced by acetate, since it is a natural product of the ChbG enzyme and is detected in several previous CDA and COD structural studies^20–22^. The metal ion and acetate ion were localized at the cavity formed around the putative active site of kpChbG, and the distance between the two ions was 6.1 Å (Fig. 3b). The acetate ion was coordinated by L13, L66, S217, and R223. Residues D10 and H206 might be responsible for kpChbG deacetylation activity by working as a general base and acid, respectively, since they were also localized at the putative active site (Fig. 3c). The metal ion was identified as Zn^2+^ by inductively coupled plasma mass spectrometry (ICP-MS)^23^ with a detected concentration of 701.7 ppb (µg/kg) in around 30 µM of ChbG protein (Fig. 3d). This correlated with previous studies of CDAs and CODs, showing that zinc was the major metal ion detected and is critical for deacetylase activity^19,24–26^.

### Structural comparison of kpChbG with its structural homologs

The proposed molecular mechanism of diacetylchitobiose deacetylation was investigated by comparison of dimeric kpChbG to its structural homologs using the Dali server^27^. The two most similar proteins (highest Z-score and RMSD) were hypothetical protein EF3048 (PDB 2I5I), having a 35% sequence identity with kpChbG, and hypothetical protein TTHB029 (PDB 2E67)^16^ (Table 2). Hypothetical protein TTHB029 from *Thermus thermophilus* shares 22% sequence identity with kpChbG and might be its homolog. This is the only solved structure which closely resembles ChbG. Two other peptidoglycan deacetylase structures from *Streptococcus pneumoniae* (PDB 2C1G)^18^ and *Bdellovibrio bacterivorous* (PDB 5JP6)^39^ were searched as the third and fourth matches, respectively, although their low sequence identity (~12–14 %) suggests that they may not be structurally related to kpChbG (Table 2).

**Table 2:** Structural similarity search using the DALI server^27^.

Protein (accession number), species (PDB)	Z-score	RMSD (Å) (amino acids)	Identity (%)	References
Hypothetical protein (EF3048), *Enterococcus Faecalis* (2I5I)	34.6	1.6 (244)	35	unpublished
Hypothetical protein (TTHB029), *Thermus thermophilus* (2E67)	24.5	2.4 (227)	22	^16^
Peptidoglycan deacetylase, *Streptococcus pneumoniae* (2C1G)	13.1	2.7 (169)	12	^18^
Peptidoglycan deacetylase, *Bdellovibrio bacterivorous* (5JP6)	10.3	2.8 (168)	14	^39^

**Fig. 4:**
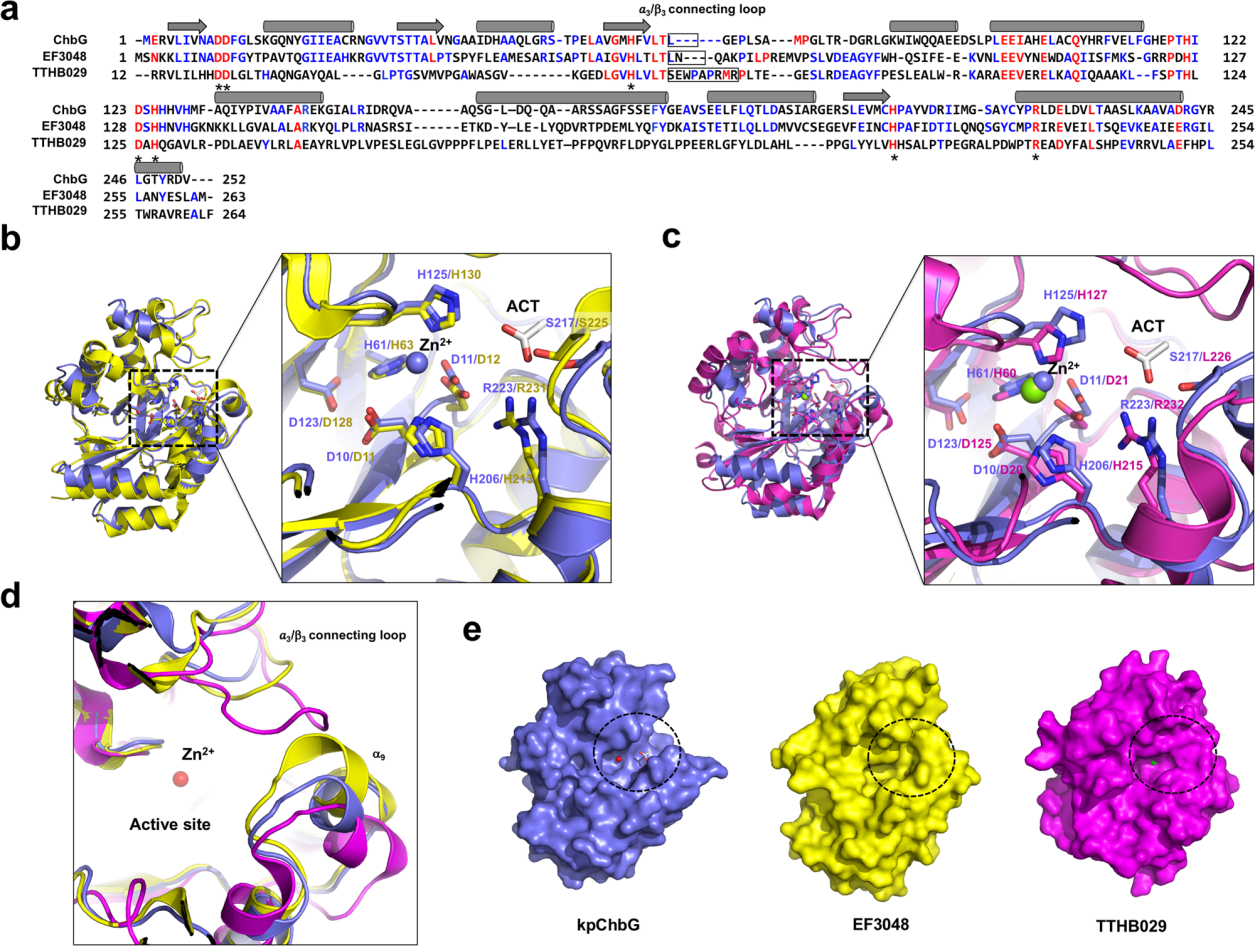
Comparison of kpChbG with its structural homologs: hypothetical proteins EF3048 and TTHB029. **a** Sequence alignment using Clustal Omega. Completely conserved and partially conserved residues are colored red and blue, respectively. The positions of the α3/β3 connecting loop and the α9 helix are indicated by black squares. Conserved residues that are involved in the formation of the putative active site are indicated by asterisks (*). Structural superposition of kpChbG (light blue) with EF3048 (yellow) (**b**) and TTHB029 (magenta) (**c**). Black-dashed boxes indicate the magnified region of the putative active site. **d** Cartoon representation showing the specific region containing the α3/β3 connecting loop and the α9 helix around the putative active site which might be critical for substrate specificity. kpChbG (light blue color), EF3048 (yellow color), and TTHB029 (magenta color) were superposed for structural comparison. **e** Overall surface feature of the three homologous proteins. The black-dashed circle indicates the ChbG active site containing the α3/β3 connecting loop and the α9 helix.

Sequence alignment indicated that all three residues involved in metal coordination in the tentative active site (D11, H61, and H125), and two amino acids which might be general acid and base residues for the deacetylation reaction (D10 and H206) were completely conserved in EF3048 and TTHB029, indicating that the hypothetical proteins, EF3048 and TTHB029, are ChbG homologs (Fig. 4a). Structural comparison of monomeric kpChbG with monomeric EF3048 and TTHB029 by superposition revealed that the tentative active site was structurally conserved by exhibiting almost identical side chain locations of the active site forming residues, although the precise structure was relatively different, with RMSD values of 1.6 Å and 2.4 Å for EF3048 and TTHB029, respectively, due to the low sequence identity (Fig. 4b, c). Although the overall kpChbG fold was similar with EF3048 and TTHB029, the position and length of several loops and the location of several helixes differed. In particular, the length and location of the α3/β3 connecting loop around the tentative kpChbG active site was completely different with kpChbG having the shortest form, whereas TTHB029 had the longest form and was positioned towards the α9 helix (Fig. 4d). The α9 helix around the active site of EF3048 was localized closer to the α3/β3 connecting loop, whereas it was far from the α3/β3 connecting loop in TTHB029 (Fig. 4d). These structural differences around the active site appear to contribute to differences in the surface features of each protein. Since kpChbG had the shortest α3/β3 connecting loop and the α9 helix was localized far from the α3/β3 connecting loop, the tentative active site of kpChbG showed an open conformation with a wider, deeper groove than observed in the other two homologs (Fig. 4e). However, EF3048 and TTHB029 exhibited closer localization of the α3/β3 connecting loop to the α9 helix, so the active site has a more closed conformation by having a narrow active site (Fig. 4e). The size control of the active site by the α3/β3 connecting loop and the α9 helix might be important for the substrate specificity.

### Structural comparison of kpChbG with other CE4 enzyme family members

**Fig. 5:**
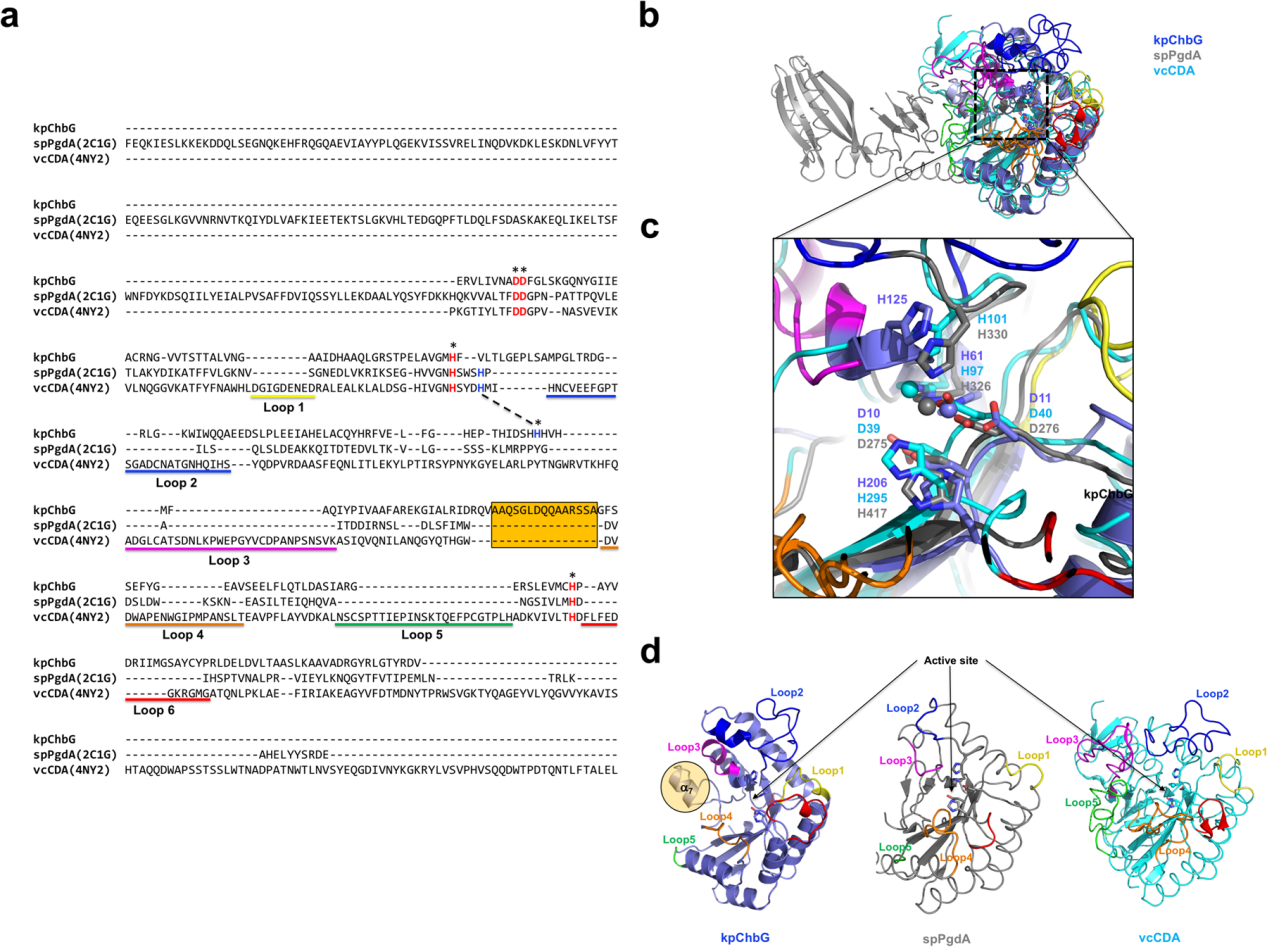
Comparison of kpChbG with other CE4 enzyme family members. **a** Sequence alignment based on structural alignment using PROMALS3D. The five residues forming the active site, which are critical for activity of CE4 enzymes, are indicated by asterisks (*). Four of the five residues forming the active site structurally aligned with each other are shown in red, whereas one residue, H125 on kpChbG, which does not structurally aligned, is shown in blue. The putative corresponding histidine residue with H125 of kpChbG, identified by locating the similar position in the active site of the CE4 enzyme, is linked by a dashed black line. Six loops (Loop 1~6) involved in the substrate specificity control as characterized in CE4 enzymes are indicated by colored lines under the corresponding residues. Residues used to form α7 helix in kpChbG are highlighted using orange color. **b** Structural superposition of kpChbG (light blue) with spPgdA (gray) and vcCDA (cyan). **c** Magnified region of the active site that is marked by a black-dashed box in **b**. Conserved residues involved in the formation of the active site are labeled. **d** Structural comparison of six loops in kpChbG with those of other CE4 enzymes. α7 helix, which is only present in kpChbG, is indicated by an orange-colored circle.

Considering functional similarity, the structure of kpChbG was compared with other CE4 enzyme family members. The selected representative peptidoglycan deacetylase and chitin deacetylase used for structural comparison with kpChbG were PgdA from *Streptococcus pneumoniae* (spPgdA) (PDB id: 2C1G)^18^ and CDA from *Vibrio cholerae* (vcCDA) (PDB id: 4NY2)^20^, respectively. The comparative sequence identities were very low, with kpChbG having a 12 % identity with spPgdA and an 8% identity with vcCDA (Fig. 5a). Sequence alignment, based on structural alignment using PROMALS3D, indicated that four residues (D10, D11, H61, and H206) among five in the active site were completely conserved in spPgdA and vcCDA (Fig. 5a). Residue H125 of kpChbG, which is involved in the metal ion coordination, was not conserved in both CE4 enzyme family members. Structural comparison of monomeric kpChbG with spPgdA and vcCDA by superposition showed low structural similarity between kpChbG and either CE4 enzyme, with RMSD values of 2.7 Å and 3.8 Å for spPgdA and vcCDA, respectively (Fig. 5b). Interestingly, however, the tentative active site of kpChbG is structurally conserved with other CE4 enzymes as supported by the presence of almost conserved residues in the active site and almost identical side chain locations of the active site forming residues (Fig. 5c). In addition, structural comparison revealed that the location of H125 in kpChbG was overlapped by H330 in spPgdA and H101 in vcCDA, indicating that H125 in kpChbG structurally aligned with the same histidine residue in other CE4 enzymes. Since H330 in spPgdA and H101 in vcCDA are considered important residues for metal coordination, we concluded that H125 in kpChbG, while not sequentially conserved by structurally aligned, is also used for metal coordination.

Although the active site kpChbG fold was similar to that of other CE4 enzymes, the position and length of six loops, which are known to be involved in the substrate specificity control in other CE4 enzymes^18,20,28^, were quite different (Fig. 5d). In particular, the length and location of loop 6 of kpChbG was completely different from that of other CE4 enzymes: kpChbG exhibited the longest form, whereas loop 6 of spPgdA exhibited the shortest form (Fig. 5d). In turn, vcCDA exhibited the longest loop 2, 3 and 4. Interestingly, the α7 helix of kpChbG, which is surrounded by loop3, 4, and 5, was only detected in kpChbG. Since the α7 helix of kpChbG was localized near substrate-determining loops, the function of this helix on the substrate specificity control should be analyzed in the near future.

### Sequence comparison of various prokaryotic and eukaryotic ChbG homologs

**Fig. 6:**
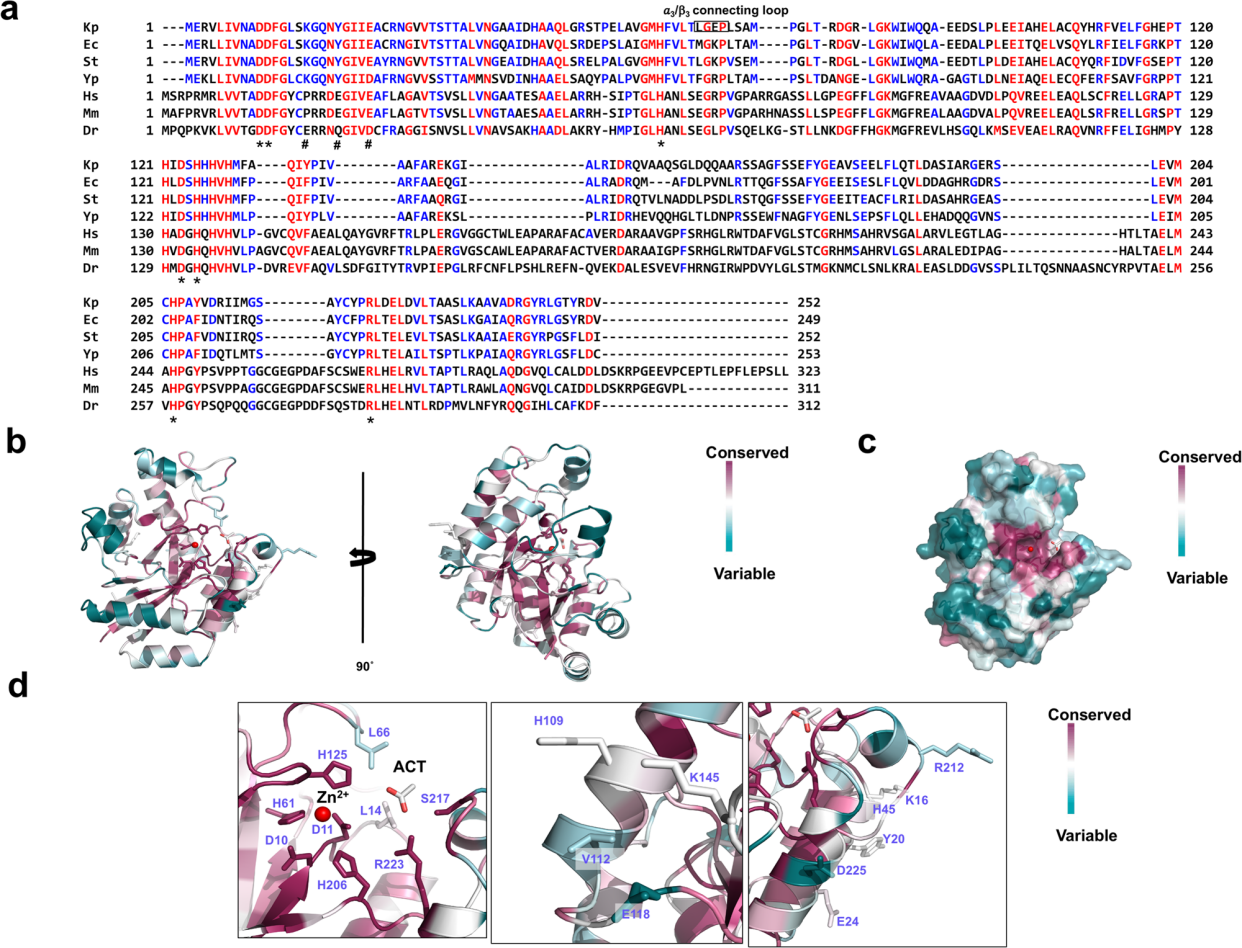
Sequence comparison of prokaryote and eukaryote ChbG homologs. **a** Sequence alignment using Clustal Omega Completely conserved and partially conserved residues are shown in red and blue, respectively. The position of the α3/β3 connecting loop is indicated by a black square. Conserved residues that are involved in the formation of the putative active site and the A/B’ dimer are indicated by asterisks (*) and hashes (#), respectively. Kp: *Klebsiella pneumoniae*, Ec: *Escherichia coli*, St: *Salmonella typhimurium*, Yp: *Yersinia pestis*, Hs: *Homo sapiens*, Mm: *Mus musculus*, Dr: *Danio rerio*. **b** Cartoon representation of kpChbG colored according to the degree of amino acid sequence conservation generated by the ConSurf server^29^. **c** Surface representation of kpChbG. **d** Three close-up views of panel (**b**) showing the amino acids of the putative active site, the first dimer PPI, and the second dimer PPI.

ChbG is a unique COD that is conserved in bacteria to mammalian genomes. The general working mechanism of this family was investigated by aligning the sequences of several representative ChbGs from bacterial, mammalian species, and fish species. Bacterial and mammalian ChbG are composed of approximately 250 and 310 residues, respectively. The amino acids involved in the formation of the putative active site are completely conserved across the species, although the overall sequence identity is relatively low ~20% (Fig. 6a). The ConSurf server^29^ showed that the putative ChbG active site (metal and acetate) residues, and those around it were the most evolutionarily conserved, indicating that the deep groove formed by D10, D11, H61, H125, H206, and R223 is the kpChbG substrate binding site (Fig. 6b, c). Interestingly, the kpChbG dimer formation residues (K16, Y20, and E24) were only conserved in bacterial ChbG, indicating that mammalian ChbG might not form a dimer, and ChbG dimerization may be exclusive to bacterial species (Fig. 6a, d).

### Model of diacetylchitobiose deacetylation by kpChbG

**Fig. 7:**
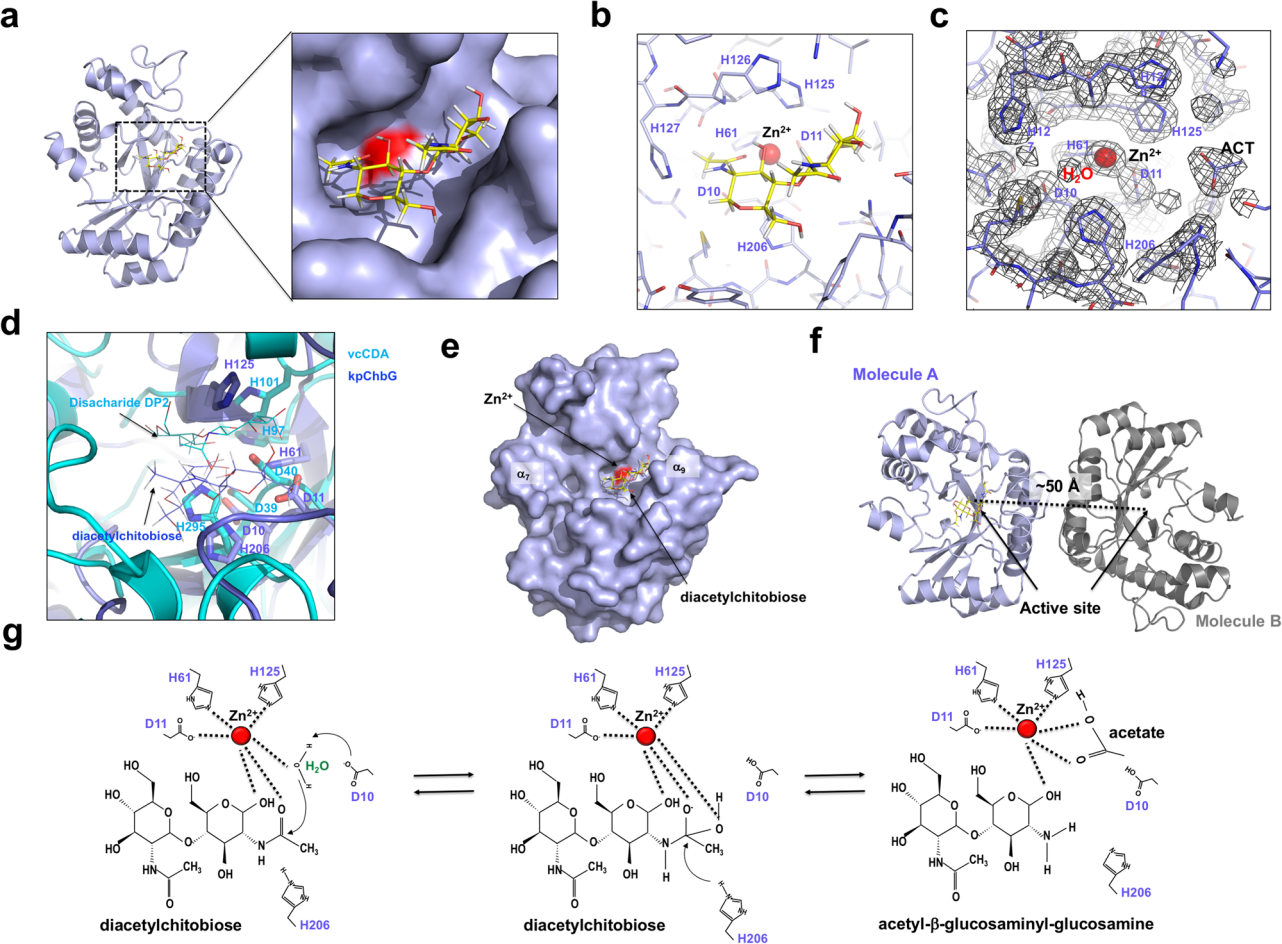
Proposed model of diacetylchitobiose deacetylation mechanism by kpChbG. **a** Cartoon representation of diacetylchitobiose substrate docking into the putative active site of kpChbG. Magnified view of docking site in the surface figure is provided. **b** Close-up of the kpChbG active site after diacetylchitobiose docking. **c** The electron density map around the active site of kpChbG. The 2Fo-Fc density map contoured at the 1σ level is shown. **d** Structural superposition of kpChbG/diacetylchitobiose complex model (blue color) with vcCDA/disaccharide DP2 complex (Cyan color). The location of two substrates are indicated by black arrows. Five core residues at the active site were labeled. **e** Surface representation of diacetylchitobiose docking in the putative active site of kpChbG. The locations of α7 helix and α9 helix are indicated on the surface. Red-colored surface indicates Zn^2+^ ion binding region. **f** The final dimeric model of kpChbG with bound substrate in the active site. **g** Proposed Zn^2+^-assisted enzymatic mechanism of kpChbG.

Diacetylchitobiose was docked into the kpChbG structure using the GLIDE docking package in Schrödinger-Maestro version 13.1 released at 2022 to demonstrate the possible substrate binding and working mechanism of the enzyme. The best docking molecule has −6.2 glide score with Gibbs free energy (ΔG) value of −12.33 kcal/mol. The most energetically favorable substrate binding molecule with kpChbG picked by docking program fitted well into the deep putative active site cavity formed by six active site residues (Fig. 7a, b) and localized to the upper side of the metal ion (Fig. 7b, c). The docked acetyl group of diacetylchitobiose localized to the deep cavity around Zn^2+^ ion almost where the opposite site as that found in the crystal structure (Fig. 7a–c). A potentially nucleophilic water molecule triggering deacetylation was detected adjacent to Zn^2+^ (Fig. 7c).

We next sought to better understand the correct substrate binding to kpChbG and to compare the substrate recognition strategy of kpChbG with that of other CE4 enzymes. To accomplish this, we used structural superposition to compare our docking model findings with that of selected representative structures of the substrate bound CE4 enzyme vcCDA (PDB id: 4NY2). This comparison showed that the overall substrate binding position of both enzymes was similar although the substrate binding in vcCDA was localized a little more to the upper side (Fig. 7d). This indicated that kpChbG and CE4 enzymes may use the same strategy for accommodating the substrate using the five core residues conserved in the active site.

Since diacetylchitobiose-6-phosphate is also expected to be a substrate for kpChbG, we performed the docking study using this substrate as well to rationalize the specificity of the enzyme. The analysis showed that the 6-phosphate group localized near the Zn^2+^ ion participating in Zn^2+^ coordination (Supplementary Fig. 4). Although this location was not expected, the position of 6-phosphate group was similar with the position of the acetyl group supposed to be cut out of docked diacetylchitobiose supposed to be cut out. These docking results may indicate that diacetylchitobiose-6-phosphate might use different way to be cut acetate group by ChbG.

Based on our docking model, we analyzed the position of α7 helix, which was only detected in kpChbG, and α9 helix, whose location was variable among the homologs. This analysis showed that α7 helix and α9 helix were localized at both ends of the putative substrate binding pocking (Fig. 7e), indicating that both helixes might be involved in substrate specificity control by adjusting the length of the substrate. The distance between the two identical active sites in the dimeric kpChbG structure was approximately 50 Å (Fig. 7f).

Based on the docking and structural analysis, we finally proposed a model of mechanism of kpChbG catalysis (Fig. 7g). Following substrate binding to the active site of kpChbG, Zn^2+^ coordination aids in its correct positioning. Zn^2+^ coordination polarizes the carbonyl amide of diacetylchitobiose which reacts with an activated (nucleophilic) water molecule adjacent to Zn^2+^ to form a tetrahedral oxyanion intermediate. Water is activated by D10 working as a general base. H206 acts as a general acid and protonates the nitrogen group by facilitating C-N bond breakage, resulting in the formation of acetate and acetyl-glucosaminyl-glucosamine products. It is likely that this metal and water-dependent enzymatic mechanism is conserved in mammalian ChbG because all residues involved in active site formation are conserved across the species. In our proposed model, since D10, H61, and H206 are critical for the ChbG activity, we finally confirmed our model by mutagenesis followed by activity analysis. To perform this test, D10, H61, and H206 were mutated to alanine, generating D10A, H61A, and H206A mutants, and purified those mutants using the same method used for purification of wildtype kpChbG. By unknown reason, however, D10A was not expressed in our bacterial system, while H61A and H206A mutants was sufficiently well purified for biochemical study. Then, the deacetylation activity of prepared H61A and H206A mutants was measured by ESI-MS. As expected, these measurements showed that both mutants failed to produce the product when N,N’-diacetylchitobiose used as substrate (Supplementary Fig. 5a, b), confirming that our proposed model might be convincing. Although our docking model was well matched with proposed enzymatic mechanism of kpChbG, the location of produced acetate is still enigmatic. To answer this question and to avoid any uncertainty of this molecular- docking based mechanism study, the complex structure between enzyme and substrate (or product) should be determined in near future.

## Conclusions

In summary, this study provided the high resolution ChbG crystal structure from *K. pneumoniae* with zinc and acetate binding to distinct amino acid resides designated as the active site. Understanding the ChbG catalytic mechanism from *K. pneumoniae* may help to design next-generation antibiotics targeting ChbG deacetylase activity, and engineer CDAs and CODs as biocatalysts to produce various chitosan products for industrial and medicinal applications.

## Methods

### Cloning, protein expression, and purification

The gene encoding full-length *kpchbG* corresponding to amino acids 1–252 (GenBank QKK70562.1) was synthesized by BIONICS (Seoul, Republic of Korea). The expression plasmid was constructed by inserting the synthesized gene product into a pET28a vector digested at the NdeI and XhoI restriction sites. The pET28a-*kpchbG* expression vector was transformed into *Escherichia coli* BL21 (DE3) using heat shock at 42 °C, and spread out onto a lysogeny broth (LB) agar plate containing 50 µg/mL kanamycin and incubated for 20 h at 37 °C. A single recombinant colony was selected, cultured overnight at 37 °C in 5 mL LB media containing 50 µg/mL kanamycin, and then used to inoculate 2 L media. Protein expression was induced at an optical density (600 nm) of 0.6–0.7 with 0.25 mM isopropyl β-D-1-thiogalactopyranoside, and the cells were cultured for 18 h at 20 °C in a shaking incubator. Subsequently, bacterial cells were harvested by centrifugation at 3000 *g* for 20 min at 4 °C, and the cell pellet was resuspended in 16 mL lysis buffer [20 mM Tris-HCl pH 8.0, 500 mM NaCl, and 25 mM imidazole]. After addition of 20 mM phenylmethanesulfonyl fluoride (Sigma-Aldrich, St. Louis, USA), the cells were disrupted by sonication on ice with twelve bursts of 30 sec each and a 30 sec interval between each burst using a sonicator. The cell lysate was centrifuged at 10,000 *g* for 30 min at 4 °C. The supernatant was collected and mixed with nickel-nitrilotriacetic acid (Ni-NTA) resin (Qiagen, Hilden, Germany) by gentle agitation overnight at 4 °C. The supernatant/Ni-NTA resin mixture was poured into a gravity-flow column and washed with 100 mL wash buffer [20 mM Tris-HCl pH 8.0, 500 mM NaCl, and 30 mM imidazole] to remove unbound proteins. Purified protein was eluted using 3 mL elution buffer [20 mM Tris-HCl pH 8.0, 500 mM NaCl, and 250 mM imidazole]. The eluate was concentrated to 30 mg/mL using a 10 kDa cut off centrifugal filter unit (Sigma-Aldrich) and purified by SEC with an ÄKTA explorer system (GE Healthcare, Chicago, USA) using a 24 mL Superdex 200 Increase 10/300 GL column (GE Healthcare) pre-equilibrated with SEC buffer [20 mM Tris-HCl pH 8.0 and 150 mM NaCl] at a flow rate of 0.4 mL/min and 4 °C. The peak fractions were pooled, concentrated to 5 mg/mL, flash-frozen in liquid nitrogen, and stored at −80 °C until use. Protein purity was assessed using SDS-PAGE.

### Deacetylation assay by electrospray ionization mass spectrometry

The reaction mixture (100 µL) containing 20 mM Tris-HCl buffer (pH 8.0), 10 mM substrate (N,N’-diacetylchitobiose), and 12.8 µM purified enzyme was incubated for 4 h at 37 °C with shaking at 800 rpm. The wild type purified enzyme was boiled for 15 min and the control was analyzed using similar conditions. The reaction was stopped by boiling the mixture for 10 min. Thereafter, the clear supernatant was collected and subjected to the ESI-MS analysis. The samples were analyzed using SYNAPT G2 (Waters, U.K.) HR ESI-MS instrument.

### Crystallization and data collection

Initial crystal screening involved the hanging drop vapor diffusion method by mixing 1 µL of 5 mg/mL kpChbG with an equal volume of reservoir solution (0.1 M MES pH 6.5 and 15% (v/v) polyethylene glycol 550 monomethyl ether (PEG550MME))and equilibrating against 300 µL of the mother liquor at 20 °C. Crystals were obtained after three days. The crystallization conditions were further optimized to a buffer composition of 0.1 M MES pH 6.65 and 16% (v/v) PEG550MME. Diffraction-quality crystals appeared in five days and grew to a maximum size of 0.1 × 0.1 × 0.2 mm^3^. For data collection, the crystals were soaked in the mother liquor supplemented with 40 % (v/v) glycerol as a cryoprotectant, mounted, and flash-frozen in a nitrogen stream at −178 °C. The diffraction data were collected at the Pohang Accelerator Laboratory (PAL) with the 5 C beamline (Pohang, Republic of Korea) at a wavelength of 1.000 Å. The diffraction data were indexed, integrated, and scaled using the HKL-2000 program (HKL Research, Inc)^30^.

### Structure determination and analysis

The structure was determined by molecular replacement using Phaser^31^. The EF3048 hypothetical protein structure (PDB 2I5I, deposited but unpublished) was used as the search model since it has 35% amino acid sequence homology with kpChbG. The initial model was constructed using AutoBuild in Phenix and completed with Coot^32^. Model refinement was iteratively performed using phenix.refine in Phenix^33^. The quality of the model was validated using MolProbity^34^. All the structural figures were generated using the PyMOL program^35^.

### Mutagenesis

Site-directed mutagenesis was conducted using a Quick-change kit (Stratagene) according to the manufacturer’s protocols. Mutagenesis was confirmed by sequencing from BIONICS (Seoul, Republic of Korea). Mutant proteins were prepared using the method described above.

### SEC-multi-angle light scattering (MALS) analysis

kpChbG (5 mg/mL) was filtered with a 0.2 µm syringe-filter and loaded onto a Superdex 200 10/300 column attached to the ÄKTA explorer system (GE Healthcare) pre-equilibrated in SEC buffer and analyzed with a DAWN-Treos MALS detector (Wyatt Technology, Santa Barbara, USA). Samples were eluted at a flow rate of 0.4 mL/min at 25 °C. The absolute molecular mass of kpChbG was assessed using the ASTRA program (Wyatt Technology) with the molecular mass of bovine serum albumin used as a reference.

### Sequence alignment

ChbG amino acid sequences from various species were analyzed using Clustal Omega (http://www.ebi.ac.uk/Tools/msa/clustalo/).

### Inductively coupled plasma-mass spectrometry (ICP-MS) analysis

The trace metal ion concentration in kpChbG was determined by comparative analysis of a serial dilution of the Recipe® control samples (Munich, Germany) prepared in water. A mixture of Be and Co internal standard was added to all calibration points and samples at specific concentrations. The measurements were performed by a NexION350D ICP-MS (Perkin-Elmer SCIEX model) using an Argon plasma source at the National Center for Inter-University Facilities in Seoul National University (Seoul, Korea). The sample was injected at 1.00 mL/min. The data shown are the mean of triplicate samples.

### Molecular docking analysis

Ligand file in PDB format derived from PDB databank (www.rcsb.org) was converted to mol format using the Open Babel tool (http://openbabel.org/wiki/Main_Page)^36^. Ligand was prepared by using “LigPrep” in the package of Schrödinger-Maestro version 2022-1 using default parameter^37^. The target protein, solved structure of kpChbG, was prepared by “protein preparation wizard” in GLIDE package of Schrodinger-Maestro^37^. Charges and bond orders were assigned, and hydrogens were added in the protein molecule during the protein preparation steps. The standard protonation state of residues at physiological pH 7.4 was applied to all residues. Water molecules were removed, and hydrogen atoms and missing side chains were automatically added using the CHARMM-based target preparation system^38^. Finally, the energy-minimization process was performed on 100 steps of the steepest descent algorithm to generate ready-to-go target receptors. Substrate ligand docking to kpChbG was performed using glide standard (SP) ligand docking program in GLIDE of Schrödinger-Maestro version with default input parameters^37^. Total binding energy and binding pose of the ligand to specific regions of kpChbG were carefully examined, and the best scored docking molecule with lowest GLIDE score value was selected for further analysis using PyMOL.

### Reporting summary

Further information on research design is available in the Nature Research Reporting Summary linked to this article.

## Supplementary information

Peer Review File

Supplementary Information

Reporting Summary

## Data Availability

Atomic coordinates and structure factors for the reported crystal structures have been deposited in the Protein Data bank (accession number 7VI8).
