# Supplementary material for: Crystal structure of ChbG from Klebsiella pneumoniae reveals the molecular basis of diacetylchitobiose deacetylation
Source: Commun Biol. 2022 Aug 24;5:862. doi: 10.1038/s42003-022-03824-9 (PMC9402603; doi:10.1038/s42003-022-03824-9)
Supplement: Supplementary file 2 — Supplementary Information [file 42003_2022_3824_MOESM2_ESM.pdf]

Crystal structure of ChbG from *Klebsiella pneumoniae* reveals the molecular basis of diacetylchitobiose deacetylation

So Yeon Lee[1,2], Bashu Dev Pardhe[3], Tae-Jin Oh[3,4,5], and Hyun Ho Park[1,2,*]

[1]College of Pharmacy, Chung-Ang University, Seoul 06974, Republic of Korea

[2]Department of Global Innovative Drugs, Graduate School of Chung-Ang University, Seoul 06974, Republic of Korea

[3]Department of Life Science and Biochemical Engineering, Sunmoon University, Chungnam 31460, Republic of Korea

[4]Department of Pharmaceutical Engineering and Biotechnology, Sunmoon University, Chungnam 31460, Republic of Korea

[5]Genome-based BioIT Convergence Institute, Chungnam 31460, Republic of Korea

[*]**Corresponding author:**

Hyun Ho Park; College of Pharmacy, Chung-Ang University, Dongjak-gu, Seoul 06974, Republic of Korea; Tel: +82-2-820-5930; Fax: +82-2-820-3033; Email: xrayleox@cau.ac.kr

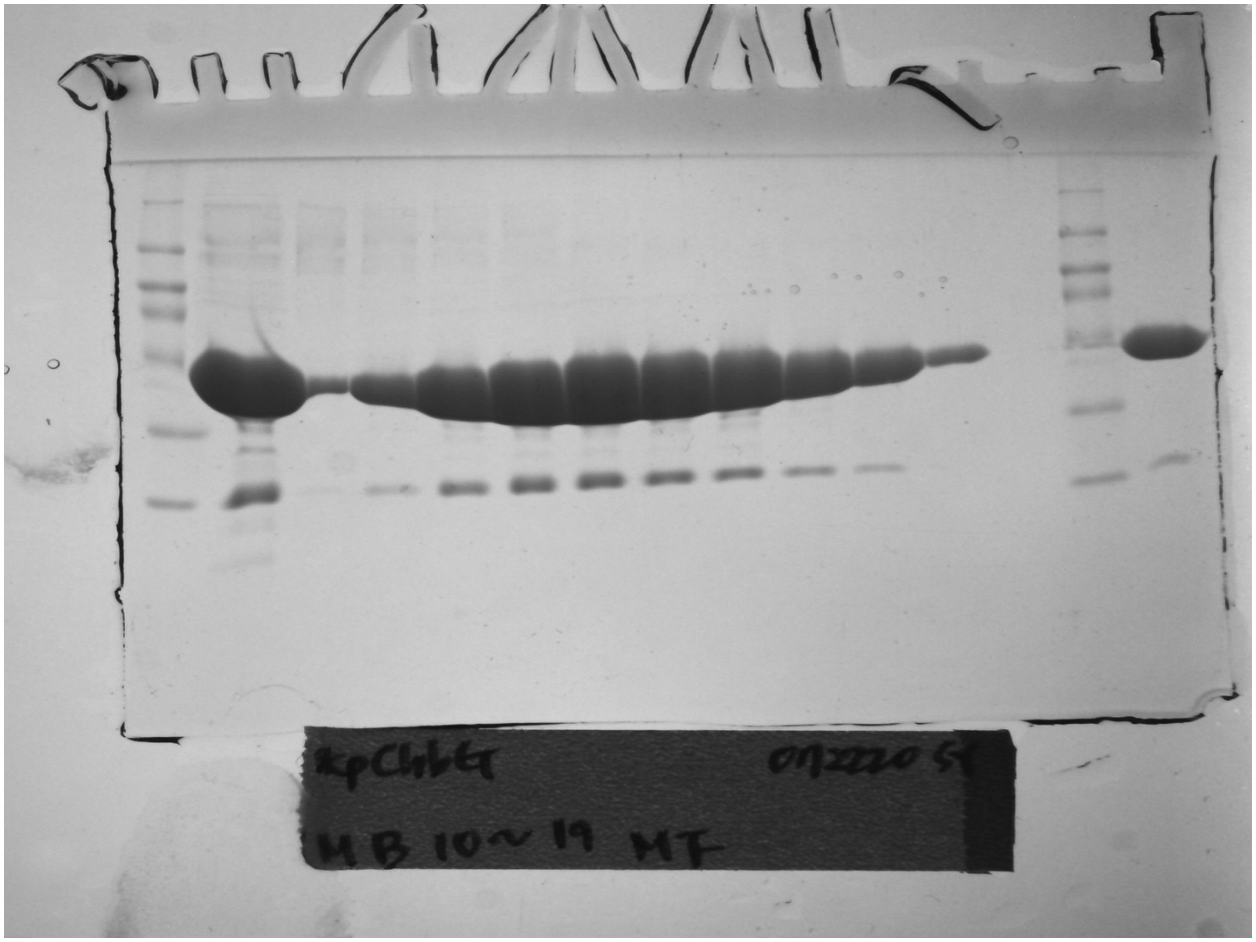

Supplementary Figure 1.
Uncropped SDS-PAGE gel used at fig1b.

a

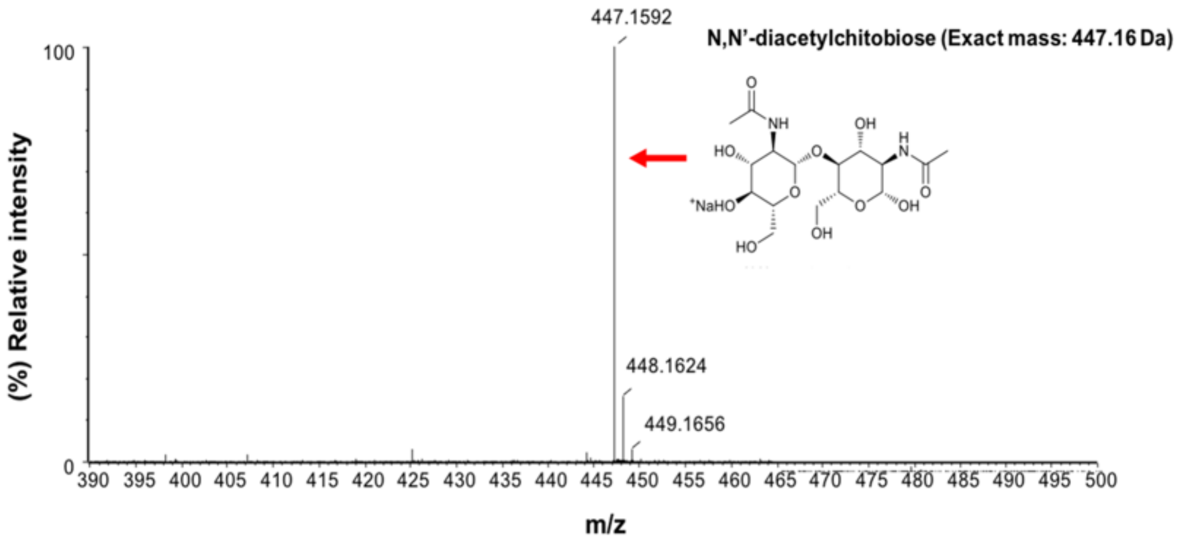

b

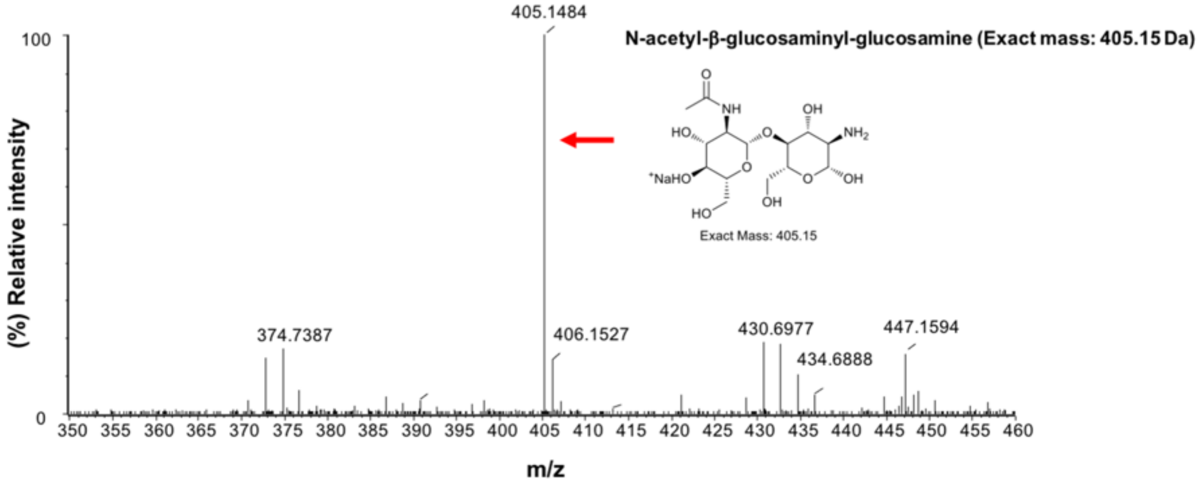

Supplementary Figure 2.
ESI-MS analysis of N,N'-diacetylchitobiose (substrate) (a) and N-acetyl-β-glucosaminyl-glucosamine (product) (b) to determine the exact molecular mass and to be used for control.

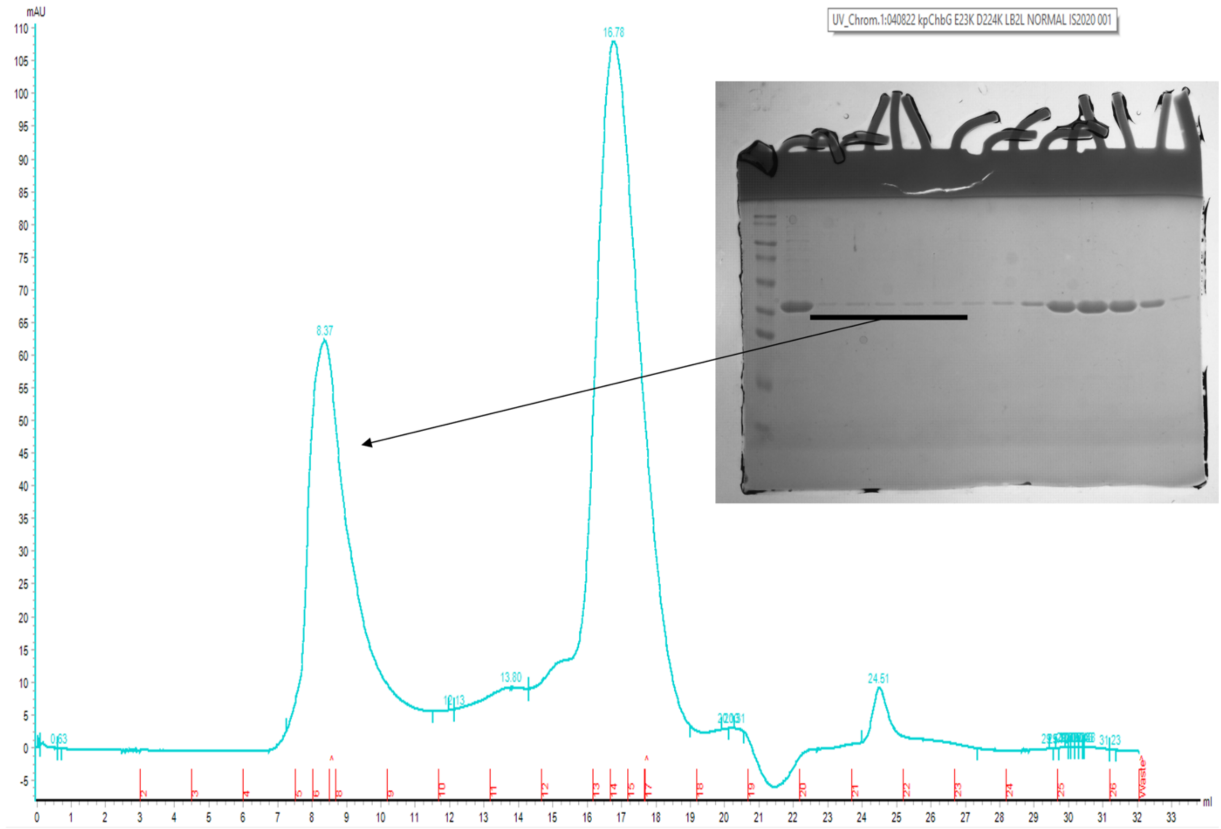

Supplementary Figure 3.
Profile size-exclusion chromatography (SEC) of E23K/D224K double mutant. SDS-PAGE gel loaded peak fractions is provided. Loaded fractions eluted around 8 mL on the SEC column are indicated by the horizontal black bar and black arrow.

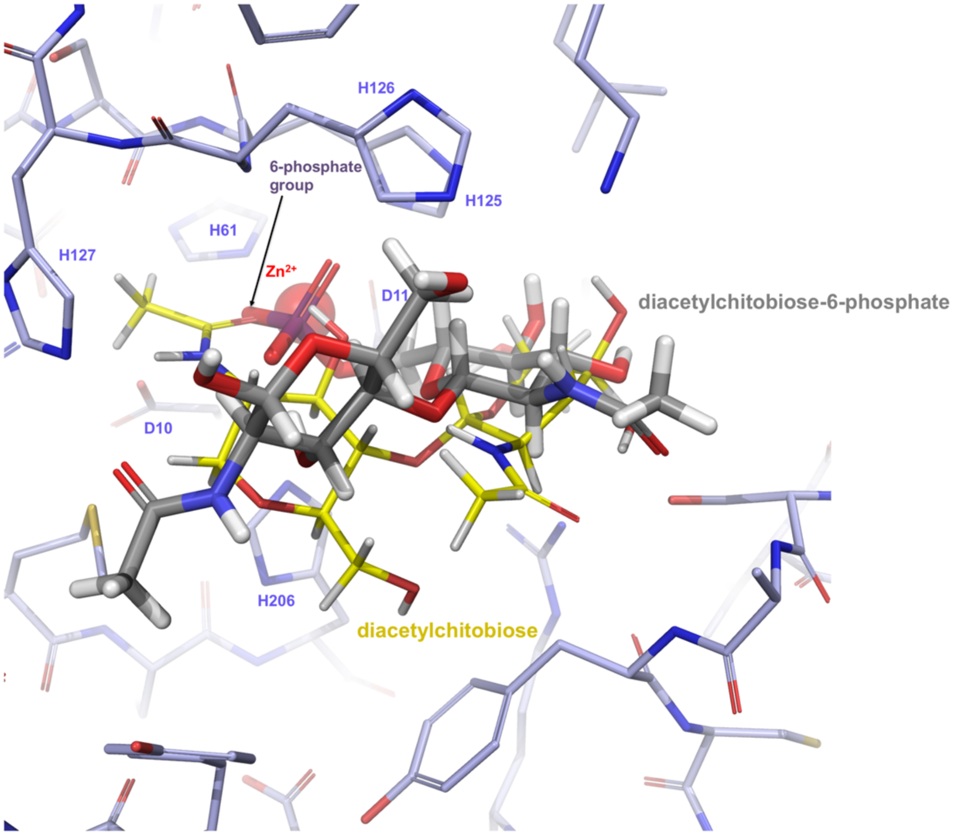

Supplementary Figure 4.
Structural comparison of kpChbG/diacetylchitobiose complex docking model with kpChbG/ diacetylchitobiose-6-phosphate complex docking model by superposition.

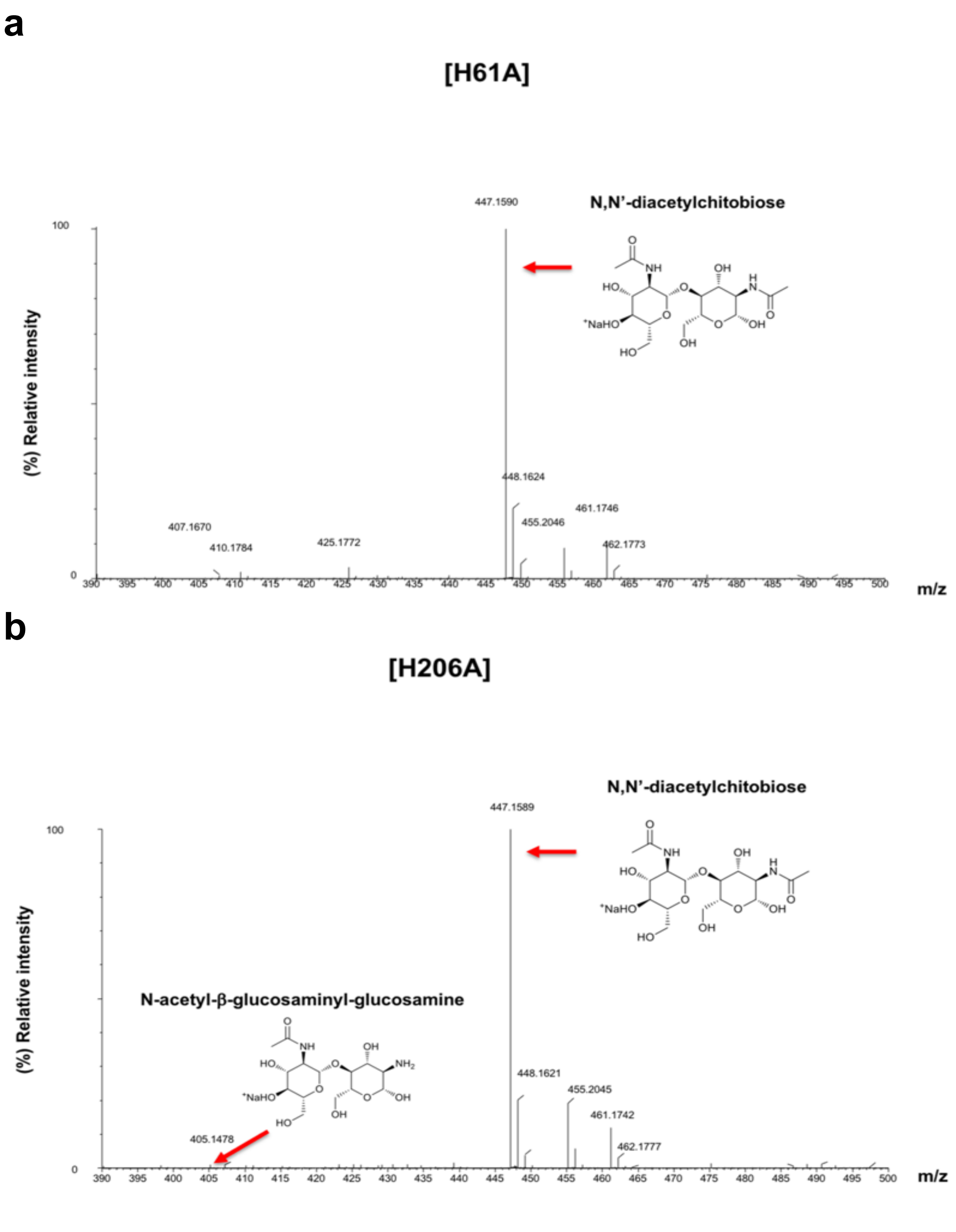

Supplementary Figure 5.
ESI-MS analysis of deacetylation of N,N'-diacetylchitobiose ($(GlcNAc)_2$) to N-acetyl-β-glucosaminyl-glucosamine (GlcN-GlcNAc) by two kpChbG active site mutants, H61A and H206A.
